# A case report of AIDS complicated by *Pneumocystis jirovecii* and *Tropheryma whipplei* mixed pulmonary infection identified via metagenomic next-generation sequencing

**DOI:** 10.3389/fmed.2025.1599261

**Published:** 2025-07-10

**Authors:** Xiaokui Sun, Qiangjin Gong

**Affiliations:** ^1^Department of Respiratory and Critical Care Medicine, Hangzhou Linping Hospital of Traditional Chinese Medicine, Hangzhou, China; ^2^Department of Respiratory and Critical Care Medicine, The First Affiliated Hospital of Anhui University of Chinese Medicine, Hefei, China

**Keywords:** *Tropheryma whipplei*, *Pneumocystis jirovecii*, AIDS, bronchoscopy, MNGs, compound sulfamethoxazole tablets

## Abstract

*Tropheryma whipplei (TW)* is a Gram-positive bacterium that is rarely encountered in clinical practice. It primarily affects the digestive and nervous systems, with pulmonary infections being infrequently reported. This case report describes a patient with HIV/AIDS who developed a mixed pulmonary infection involving *Pneumocystis jirovecii pneumonia (PJP)* and *TW*. The diagnosis was confirmed via metagenomic next-generation sequencing (mNGS) of bronchoalveolar lavage fluid obtained through bronchoscopy. Following appropriate treatment, the patient’s symptoms and pulmonary imaging findings showed marked improvement.

## 1 Introduction

Acquired immune deficiency syndrome (AIDS) patients are highly susceptible to various opportunistic infections, including complex lung infections (1). Among these patients, *PJP* is a well-recognized cause of morbidity, particularly in individuals with compromised immunity. Although *TW* is primarily associated with gastrointestinal and neurological manifestations, it can also cause pulmonary infections, albeit rarely. Co-infection with both *PJP* and *TW* is exceptionally uncommon. Only a few such cases have been reported, predominantly in severely immunocompromised individuals. For instance, Yan et al. documented a rare case of dual pulmonary infection in an AIDS patient diagnosed using mNGS, emphasizing the diagnostic complexity and clinical severity of this coinfection scenario (2). Additionally, a cross-sectional study analyzing 1,725 bronchoalveolar lavage fluid (BALF) samples using mNGS found a 4.0% positivity rate for *TW*, with only 12.9% of these patients being immunocompromised. Notably, co-infection with *PJP* was not observed in this cohort, underscoring the rarity of such dual infections (3). Despite its rarity, the potential for *TW* colonization in HIV-infected individuals has been increasingly recognized, with studies indicating a higher prevalence in bronchoalveolar lavage fluid samples from this population compared to immunocompetent individuals (4). These findings underscore the importance of considering *TW* in differential diagnoses of pulmonary infections in HIV/AIDS patients. Sulfamethoxazole-trimethoprim (SMZ-TMP) remains an effective option for treating both *PJP* and *TW* infections (5, 6), provided that no antimicrobial resistance is present. The following case report describes a rare co-infection with these two pathogens in a patient with newly diagnosed AIDS, diagnosed via mNGS.

## 2 Case presentation

**Figure 1:**
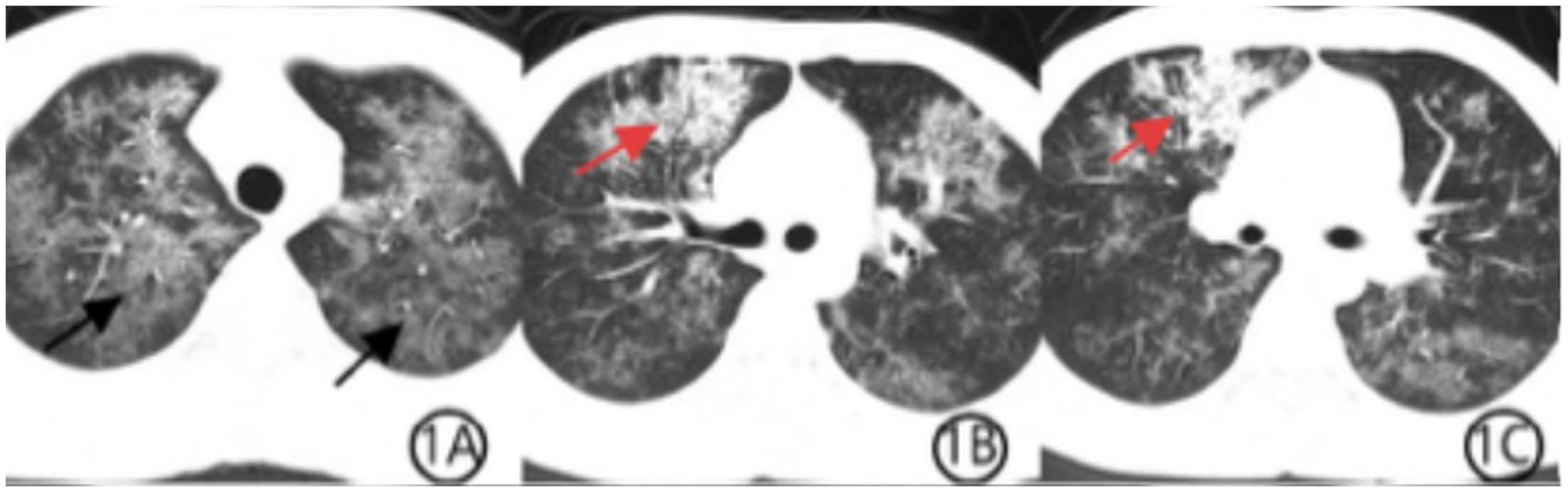
**(A–C)** Chest CT scan from 28 December 2022, at the patient’s initial presentation. Axial CT images show diffuse bilateral ground-glass opacities (black arrows), consistent with *Pneumocystis jirovecii* pneumonia. Patchy consolidation is observed in the anterior segment of the right upper lobe (red arrows), demonstrating air bronchogram signs, which are suggestive of TW infection.

We present the case of a 26-year-old married man who presented with chest tightness and wheezing lasting 1 week, accompanied by fever for 3 days. His symptoms initially began with chest discomfort, dyspnea, cough, and yellow sputum production following a recent cold. Three days prior to admission, he developed a fever peaking at 38°C, along with muscle soreness and sore throat. A chest computed tomography (CT) scan performed at a local hospital revealed multiple inflammatory lesions in both lungs (Figure 1). Upon admission, the patient was alert and in a stable condition. Auscultation revealed slightly coarse breath sounds with scattered dry and wet rales. His heart rate was 100 bpm, and his abdomen was soft and non-tender. No peripheral edema was noted. Laboratory tests showed the following abnormalities: neutrophil count 1.43*10^9^/L, reference range: 1.8–6.3 × 10^9^/L; neutrophil percentage 47.4%, reference range: 40–75%; red blood cell count 3.97 × 10^12^/L, reference range: 4.3–5.8 × 10^12^/L; hemoglobin 121 g/L, reference range: 130–175 g/L; and white blood cell count 3.02 × 10^9^/L, reference range: 3.5–9.8 × 10^9^/L; all of these results were obtained on an XN9000 hematology analyzer (Sysmex, Japan). Further results revealed a high-sensitivity C-reactive protein level of 11.2 mg/L (reference range: <1 mg/L), measured using a Remisol system (Beckman Coulter, USA). A test for fungal D-glucan was performed on an HB-100E system, showing an elevated (1-3)-β-D-glucan level of 242.04 pg./mL (reference range: <70 pg./mL). Other tests, including coagulation studies, myocardial enzyme panel, and stool analysis, were unremarkable. Serological testing for respiratory pathogens revealed borderline-positive *Mycoplasma pneumoniae* IgM. Human immunodeficiency virus (HIV) antigen/antibody screening yielded inconclusive results and was later confirmed as positive by the Hefei Center for Disease Control and Prevention (Hefei City, China).

**Figure 2:**
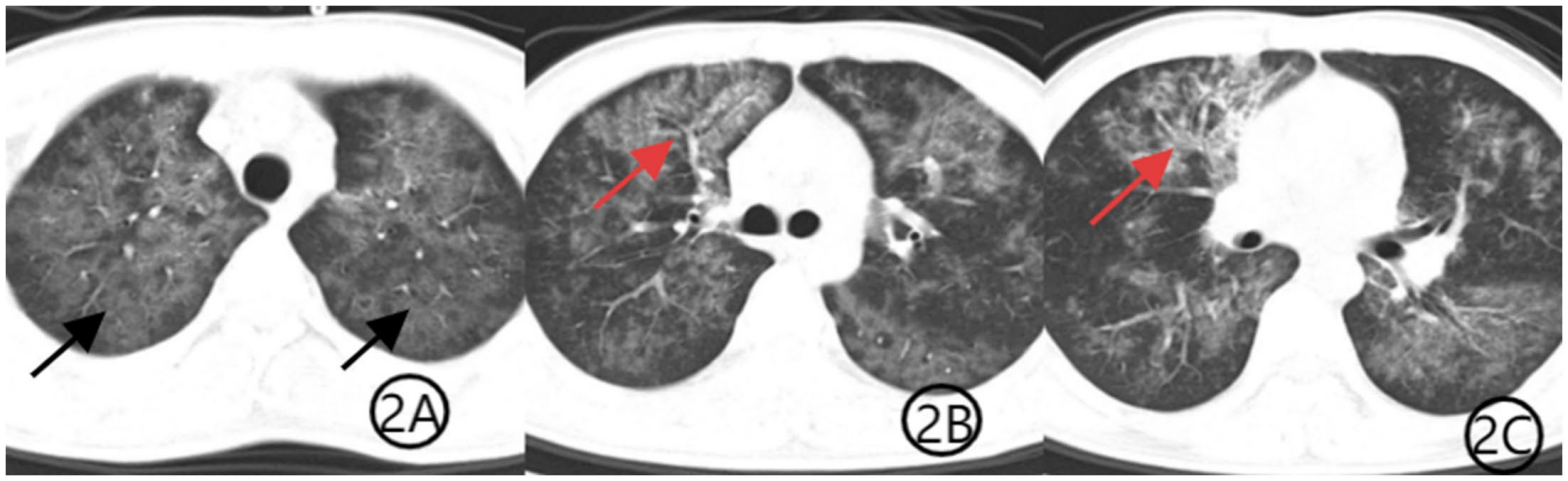
**(A–C)** Chest CT scan after 1 week of treatment. Axial CT images show improvement in bilateral diffuse ground-glass opacities (black arrows), indicating partial resolution of *PJP*. The patchy consolidation in the anterior segment of the right upper lobe is markedly reduced, with decreased visibility of the air bronchogram sign (red arrows), consistent with treatment response to TW infection.

**Figure 3:**
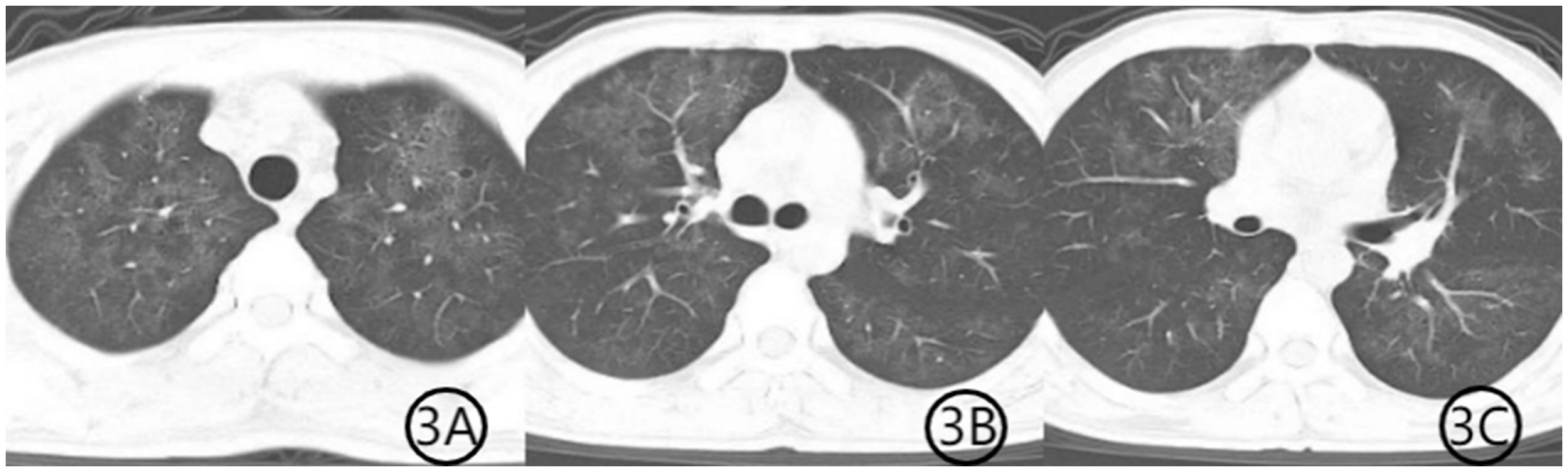
**(A–C)** Chest CT scan after 3 weeks of treatment. Axial CT images demonstrate further resolution of the previous pulmonary abnormalities. The patchy consolidation has markedly improved, the air bronchogram sign has disappeared, and the diffuse ground-glass opacities have significantly resolved, indicating substantial clinical and radiological recovery.

**Figure 4:**
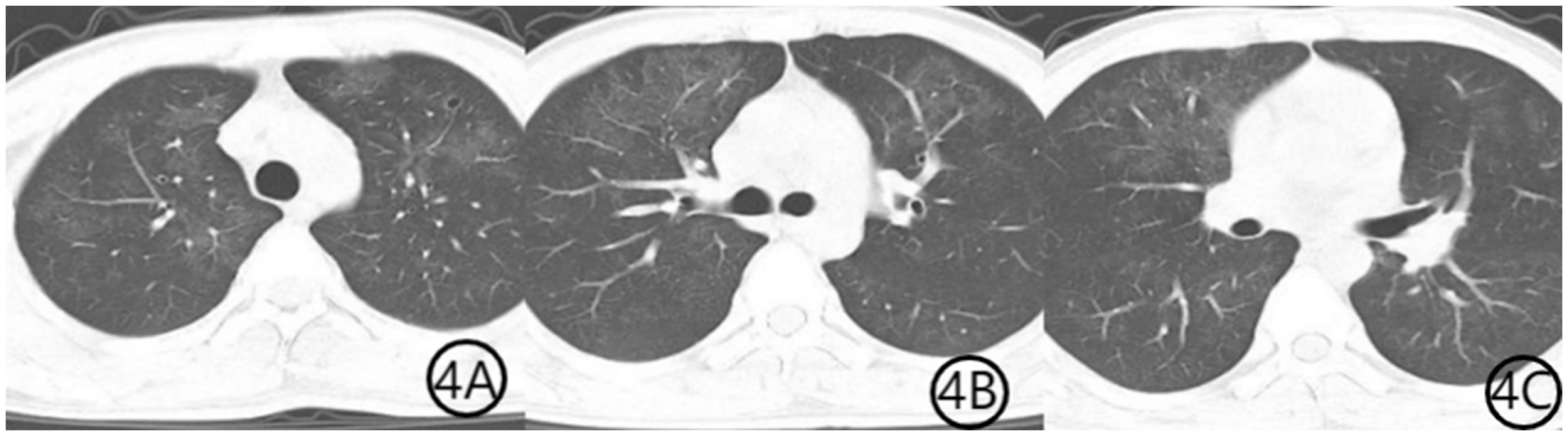
**(A–C)** Chest CT scan 1 month after initiation of treatment. Axial CT images show further resolution of bilateral diffuse ground-glass opacities, indicating continued radiological improvement and near-complete recovery from pulmonary infection.

The patient was initially diagnosed with community-acquired pneumonia and was started on intravenous levofloxacin (0.5 g qd) along with methylprednisolone (40 mg once daily) for inflammation control. Given his HIV diagnosis and clinical presentation, *PJP* was suspected. Levofloxacin was discontinued, and methylprednisolone was reduced to 20 mg after the third day of use. Oral sulfamethoxazole-trimethoprim (SMZ-TMP, 0.48 g per tablet, three tablets four times daily) was initiated, and methylprednisolone was tapered to 20 mg daily. Bronchoscopy was recommended for etiological confirmation, but the patient declined the procedure. Three days later, he opted to discontinue treatment and was discharged against medical advice. Upon discharge, he was prescribed methylprednisolone (16 mg daily), levofloxacin (0.5 g daily), SMZ-TMP (three tablets four times daily), and sodium bicarbonate (two tablets three times daily). Levofloxacin tablets were discontinued on day 10 after discharge. The SMZ dose was reduced to two tablets once daily after the third week of treatment, while methylprednisolone was reduced to one tablet every 5 days. Both SMZ and methylprednisolone tablets were taken orally until the maintenance dose of one tablet per day was taken for 5 days. One week later, the medical staff conducted a telephone follow-up with the patient and recommended that the patient undergo a repeat chest CT examination, which showed diffuse lung infection with a suspected viral etiology (Figure 2). The patient subsequently consented to bronchoscopy, which revealed no significant endobronchial abnormalities. Bronchoalveolar lavage fluid was sent for mNGS, identifying *TW* (sequence count: 23,039) and *PJP* (sequence count: 973). Three weeks into treatment, a repeat chest CT (Figure 3) showed substantial resolution of pulmonary infiltrates, and the patient’s respiratory symptoms had significantly improved. A follow-up CT scan performed 1 month later (Figure 4) demonstrated further absorption of the lung lesions.

Throughout the illness, the patient exhibited persistent chest tightness, wheezing, fever, cough, and sputum production, which were his primary complaints. Initial laboratory findings indicated leukopenia and C-reactive protein levels that were not elevated, leading to empirical treatment with levofloxacin to combat common pathogens and methylprednisolone as an anti-inflammatory agent. Upon the confirmation of HIV, *PJP* was considered a possible diagnosis, prompting the addition of SMZ-TMP (0.48 g/tablet; three tablets four times daily). Due to the absence of positive sputum cultures, bronchoscopy was advised but initially refused. After 1 week of oral SMZ-TMP, the patient’s fever resolved, and his respiratory symptoms improved. He then agreed to undergo bronchoscopy and CT re-evaluation. The latter showed improved pulmonary infiltrates, with a predominant ground-glass pattern. Bronchoscopy results revealed no obvious abnormalities. *TW* (sequence count: 23,039) and *PJP* (sequence count: 973) were detected, confirming the mixed infection. After 3 weeks of treatment, a repeat CT scan showed further improvement in pulmonary infiltrates. Levofloxacin was discontinued, methylprednisolone was tapered to 12 mg daily, and SMZ-TMP was reduced to a maintenance dose of two tablets twice daily. Four weeks later, a follow-up CT showed near-complete resolution of the infection. The patient was subsequently referred to a designated hospital for continued HIV management.

## 3 Discussion

Pulmonary infections in HIV patients, primarily due to immunodeficiency, represent a major cause of mortality. Opportunistic pathogens are frequently responsible; however, their detection using traditional diagnostic methods, such as sputum culture, sputum smear, and alveolar lavage fluid culture, remains challenging (7). This case presents a rare instance of mixed pulmonary infection with *TW* and *PJP.* Notably, the diagnosis was confirmed through mNGS performed via bronchoscopy, enabling the rapid identification of pathogens and the timely initiation of targeted therapy.

*TW* is a Gram-positive bacterium that primarily acts as an opportunistic pathogen (8). It predominantly affects the gastrointestinal, nervous, cardiovascular, and ocular systems, with pulmonary infection being infrequent (9). *TW* is widely distributed in environmental reservoirs such as sewage and sludge and can colonize the oral cavity and intestines of humans. Immunocompromised individuals, particularly those with diabetes mellitus, coronary artery disease, or a history of malignancy, are an elevated risk of *TW* infection (10). More recently, cases of *TW* infection have also been documented in patients experiencing prolonged symptoms following COVID-19 infection (11). However, infections have also been reported in immunocompetent individuals. Hao Wang described a severe case of pneumonia in a pregnant woman caused by *TW*, with subsequent analysis suggesting possible droplet transmission within her household (12). *TW* is currently recognized as being primarily transmitted via the fecal–oral and droplet—routes, while immunosuppression remains a significant risk factor for opportunistic infection (13). Studies have revealed that *TW* has a relatively high pulmonary colonization rate in healthy individuals, with even higher prevalence among HIV-positive patients (14). Upon admission, this patient was diagnosed with AIDS, a condition characterized by severe immunosuppression, which increases susceptibility to viral, fungal, and opportunistic bacterial infections. This finding aligns with prior reports describing cases of *TW* infection in immunocompromised hosts, often in conjunction with fungal co-infections (15). Stein A previously reported a case of HIV infection with low CD4 counts complicated by *TW* infection (16). Unfortunately, due to the brevity of this patient’s hospital stay, lymphocyte subset testing was not conducted. However, the results on the lymphocyte subsets were finalized during the patient’s visit to the infectious disease outpatient department at Anhui Provincial Hospital following discharge, with the report indicating CD3 levels of 540 cells/μL, CD4 levels of 19 cells/μL, CD8 levels of 497 cells/μL, and a CD4/CD8 ratio of 0.04. A CD4 count of 19 is classified as critically low, thereby rendering the individual highly susceptible to a variety of life-threatening infections. Although the CD8 level was within the normal range, it exhibited an upward trend. The inversion of the CD4/CD8 ratio reflects a profound imbalance in the patient’s immune system. The decline in CD3 can be attributed to the reduction in CD4 levels. Collectively, these lymphocyte findings indicate that the patient may have been experiencing an acute-phase HIV infection and was potentially entering a high-risk period for opportunistic infections. A study by Yuting Tan analyzing bronchoalveolar lavage fluid from 476 HIV patients and 280 non-HIV individuals found significantly higher infection rates of both *PJP* and *TW* in the HIV cohort. Notably, patients who had received antiretroviral therapy (ART) exhibited significantly lower viral and fungal titers than those who had not initiated HIV treatment (17).

*TW* is a pathogen capable of causing multisystem disease. In the gastrointestinal system, it is frequently associated with chronic diarrhea (18), and isolated organ involvement is relatively rare (19). In cutaneous infections, *TW* typically presents as subcutaneous nodules (20), while, in cardiovascular cases, it has been implicated in tricuspid valve infections (21). Pulmonary manifestations of *TW* often include cough, expectoration, dyspnea, and fever (22), which were consistent with the main complaints of this patient upon admission. Laboratory findings also revealed decreased hemoglobin, red blood cell count, and albumin levels, a pattern consistent with the findings of Lai LM, who analyzed laboratory data from 16 patients with *TW* pneumonia (23). Radiologically, *TW* pneumonia commonly presents with nodular opacities, interstitial changes, and patchy infiltrates (3). Ground-glass opacities and solid nodules are frequently reported features (24). However, previous literature lacks a consensus on the location and symmetry of pulmonary involvement, with cases reported as either unilateral or bilaterally symmetric. In this patient, a bilateral symmetrical pulmonary infection was observed, characterized by diffuse ground-glass opacities without significant pleural effusion. These radiological features, in conjunction with the patient’s clinical symptoms and laboratory abnormalities, may provide critical diagnostic clues for *TW* pneumonia.

The diagnostic challenge of *TW* infections is primarily attributed to the bacterium’s biological characteristics, including its prolonged culture cycle, susceptibility to contamination, and the difficulty of obtaining culture results through routine clinical examination methods (25). Chi Rui Bin previously reported a case of severe *TW* pneumonia in which repeated sputum and bronchoalveolar lavage fluid cultures failed to identify a definitive pathogen. However, the use of mNGS led to the successful identification of the causative agent, allowing for the timely adjustment of the treatment plan and a subsequent favorable outcome (26). Traditional diagnostic methods such as PCR, immunohistochemistry, periodic acid-Schiff (PAS) staining, and electron microscopy have been commonly used, yet lung biopsy and electron microscopy are often impractical in clinical settings. In this case, diagnosis primarily relied on mNGS of bronchoalveolar lavage fluid obtained via bronchoscopy, as mNGS is a practical and accessible diagnostic tool for clinicians. mNGS has demonstrated a significant role in the clinical diagnosis of complex pulmonary infections. Fang et al. diagnosed five cases of *TW* pneumonia through mNGS, highlighting the method’s rapid and precise detection capabilities (27). Studies utilizing mNGS on large samples of bronchoalveolar lavage fluid have revealed the presence of *TW* in the lungs of healthy individuals (28), suggesting that it may function as a colonizing bacterium. Additionally, research comparing the microbial composition of bronchoalveolar lavage fluid and oral specimens from both HIV-positive and HIV-negative individuals found that *TW* colonization was more prevalent and active in the oral cavity and lungs of HIV-positive patients (4). Reports of co-infection with *TW* and *PJP* remain scarce. In 2021, Yan documented a case involving a 28-year-old male HIV patient diagnosed with *TW* and *PJP* co-infection following mNGS testing of bronchoalveolar lavage fluid. Treatment with meropenem and SMZ led to significant resolution of the pulmonary infection (2). Similarly, Wu Dengfeng et al. reported a severe case of *TW* and *PJP* co-infection in 2023, where the patient developed respiratory failure. Treatment with meropenem, caspofungin, and SMZ for 1 week, followed by the sequential oral administration of SMZ, resulted in notable pulmonary improvement. Both HIV patients exhibited severe infections with rapid disease progression, necessitating intravenous third-line antibiotic therapy. However, in this case, the pulmonary infection did not reach a severe stage, and intravenous third-line antibiotics were not required. Instead, oral SMZ, quinolones, and methylprednisolone tablets were sufficient to control the infection effectively. A review of related cases indicates that SMZ remains a fundamental component of treatment, irrespective of disease severity (29). The management of *TW* pneumonia commonly involves antibiotics such as ceftriaxone, meropenem, sulfamethoxazole-trimethoprim, and tetracyclines. Standard recommendations suggest initial intravenous therapy with meropenem or ceftriaxone, followed by oral sulfamethoxazole-trimethoprim. However, reports of resistance to sulfamethoxazole-trimethoprim have emerged. In such cases, tigecycline combined with tetracyclines and hydroxychloroquine has been effective, suggesting an alternative therapeutic strategy for *TW* infections (30).

In this case, the high *TW* sequence count detected via mNGS, along with the patient’s clinical symptoms, strongly indicated *TW* as the primary pathogen. The role of mNGS in diagnosing *TW* pneumonia is particularly critical, given the absence of standardized treatment guidelines. HIV patients, due to their compromised immune function, are highly susceptible to secondary fungal and tuberculosis infections (31). Relying solely on conventional microbiological diagnostic methods could delay treatment, allowing pulmonary infections to progress rapidly. Fortunately, timely diagnosis and intervention in this case underscored the importance of mNGS in managing complex pulmonary infections.

Compared to *TW*, the treatment approach for *PJP* is more well-established. *PJP* is an opportunistic infection frequently observed in patients with HIV, those with malignancies, those with nephropathies, those with diabetes, and those on long-term immunosuppressive therapy. However, *PJP* has also been reported in immunocompetent individuals. The primary clinical manifestations of *PJP* include fever, dyspnea, cough, and sputum production (32). Due to the non-specificity of these symptoms, differentiation from other infections, such as *Legionella pneumonia* and *Mycoplasma pneumoniae* pneumonia, can be challenging. Additionally, atypical presentations, including chest pain, have been documented (33, 34). In this case, the patient’s fungal (1-3)-β-D-glucan level was elevated at 242.040 pg/mL (>200 pg/mL), which, in conjunction with a positive HIV test, supported a preliminary diagnosis of *PJP* (35). Prompt initiation of SMZ therapy was crucial, as it is effective against both *PJP* and *TW*. However, co-infections with SMZ-resistant bacteria could complicate treatment, highlighting the necessity for bronchoscopy and mNGS examination in cases of complex pulmonary infections.

This case report has several limitations. First, due to technical constraints in the hospital’s laboratory, CD4 count and HIV viral load testing were not performed during hospitalization. These tests were subsequently completed at the infectious disease department of a referral hospital, where a critically low CD4 count (19 cells/μL) and inverted CD4/CD8 ratio (0.04) confirmed severe immunosuppression. Second, the patient’s hospital stay was brief, primarily because he opted for early discharge while awaiting confirmatory HIV test results, citing financial considerations. Third, although bronchoscopy was recommended at admission for diagnostic clarification, the patient initially declined the procedure due to limited understanding of its necessity. Following telephone follow-up and symptom persistence, the patient agreed to undergo bronchoscopy post-discharge.

A key diagnostic challenge in this case was distinguishing between *TW* colonization and active infection. Eberhardt et al. reported a 5.85% prevalence of *TW* colonization in HIV-positive individuals in Ghana, with colonized patients showing no increased frequency of clinical symptoms compared to non-carriers. Notably, colonization was associated with better immune markers, higher CD4 counts, lower HIV viral loads, and reduced immune activation, suggesting that *TW* colonization may not be pathogenic in all HIV-positive hosts (36). In contrast, our patient presented with acute pulmonary symptoms, characteristic imaging features, and high *TW* read counts on mNGS from bronchoalveolar lavage fluid, all of which improved after targeted therapy. These factors together strongly support a diagnosis of active infection rather than incidental colonization. Recent studies have emphasized that high sequence counts in mNGS, when accompanied by compatible symptoms and radiological findings, can support the diagnosis of active *TW* pneumonia rather than colonization (3, 37). Nevertheless, the lack of a standardized diagnostic threshold for *TW* infection remains a limitation in clinical decision-making.

## 4 Summary

Immune dysfunction in HIV patients contributes to the complexity of pulmonary infections, posing diagnostic and therapeutic challenges. While clinical expertise remains essential, mNGS serves as a valuable adjunct, offering rapid and precise pathogen identification. *TW* is a rare clinical entity characterized by non-specific symptoms, a lack of distinctive imaging findings, and diagnostic challenges using conventional microbiological methods. However, immunocompromised individuals represent a high-risk group for this condition. When managing pulmonary infections in immunocompromised patients, *TW* should be considered alongside more common pathogens such as *PJP*. The use of mNGS has proven to be a powerful diagnostic tool in such cases. In this case, the patient exhibited typical imaging and clinical characteristics, and bronchoscopy combined with mNGS facilitated a timely and accurate diagnosis. Effective treatment further reinforced the importance of modern diagnostic and therapeutic approaches in managing complex pulmonary infections.

In HIV-positive patients presenting with atypical pneumonia and non-specific imaging findings, clinicians should maintain a high index of suspicion for rare opportunistic infections. Early use of mNGS in bronchoalveolar lavage fluid analysis can be instrumental in identifying uncommon pathogens and guiding targeted therapy, especially when standard microbiological tests yield inconclusive results.

## Data Availability

The clinical data referenced in this study, including bronchoscopy and mNGS test results, are available upon request. Interested parties may contact the corresponding author via email for access.
